# The Development and Impact of AYA Can—Canadian Cancer Advocacy: A Peer-Led Advocacy Organization for Adolescent and Young Adult Cancer in Canada

**DOI:** 10.3390/curroncol31050193

**Published:** 2024-05-02

**Authors:** Chantale Thurston, Julie M. Deleemans, Jason Gisser, Emily Piercell, Vinesha Ramasamy, Perri R. Tutelman

**Affiliations:** 1 AYA Can—Canadian Cancer Advocacy, Winnipeg, MB, Canada; julie.deleemans@ucalgary.ca (J.M.D.);; 2 Department of Oncology, University of Calgary, Calgary, AB T2S 3C3, Canada

**Keywords:** adolescent and young adult cancer, AYA, patient advocacy, patient and public involvement, empowerment

## Abstract

Adolescents and young adults (AYAs; 15–39 years) diagnosed with cancer face disparities in outcomes and survival. Patient advocacy organizations can play a pivotal role in advancing outcomes for underserved health conditions, such as AYA cancer. In 2018 a group of AYA patient advocates founded AYA Canada (later renamed to “AYA Can—Canadian Cancer Advocacy”), a peer-led national organization aimed at improving the experiences and outcomes of Canadian AYAs affected by cancer. The aim of this article is to describe the development and impact of AYA Can. AYA Can was incorporated as a not-for-profit organization in 2021 and became a registered charity in 2023. Since 2018, AYA Can has established a thriving community of practice comprising nearly 300 patients, healthcare providers, researchers, and charitable organizations with an interest in advocacy for AYA cancer. Other activities have included advocacy at academic conferences and on scientific committees, collaboration with scientists to advance AYA cancer research, training the next generation of AYA patient advocates through a “patient ambassador program,” and developing a national resource hub to centralize knowledge and information on AYA cancer. Through its work to foster collaboration and amplify patient priorities on a national scale, AYA Can has become a leading voice for AYA cancer advocacy in Canada.

## 1. Introduction

The incidence of cancer in adolescents and young adults (AYAs; 15–39 years) is on the rise, with an estimated 9000 AYAs diagnosed with cancer in Canada in 2023 [1]. AYAs are a distinct oncology population and face unique challenges within the cancer care system. While receiving a cancer diagnosis is an inherently difficult experience for patients of any age, AYAs must navigate the physical impact of cancer during a pivotal stage of life that is characterized by a focus on education and employment goals, romantic, social, and family relationships, fertility and family planning, and identity exploration [2,3]. AYAs exist at the margins of pediatric and adult cancer care, and neither system is adequately equipped to address the needs of this population [4]. As a result, AYAs experience significant unmet needs [5,6] and poorer psychosocial and medical outcomes compared to other age groups [7,8].

Often referred to as cancer’s “forgotten generation” [9], AYAs experience health disparities across the cancer continuum. Survival rates for AYAs have not kept pace with improvements achieved for cancers in children and older adults [7] due in part to systemic factors such as delays in diagnosis, inadequate accrual of AYAs into clinical trials, and lack of funding and research for AYA cancers [3,10,11]. AYA cancer survivors, who, given their age, will live for several decades beyond their diagnosis, face disproportionately greater medical, psychosocial, and financial impacts compared to older adult survivors [12]. While recent years have seen the establishment of a handful of AYA-specific programs in Canada [13,14,15,16], access to these services is limited based on patient geography, and significant gaps in comprehensive, evidence-informed AYA cancer care remain. Advocacy is needed to advance outcomes for Canadian AYAs diagnosed with cancer [17].

Patient advocacy organizations—defined as not-for-profit groups comprised of people with lived experience of a health condition [18]—have emerged as key players in the advancement of health research and care, particularly for rare and underserved populations. To date, patient advocacy organizations have made significant contributions to health research and care in various ways. These include raising public awareness about certain health conditions, facilitating communication between scientific and lived experience experts, democratizing scientific knowledge for the patient community to support advocacy efforts, approaching government and philanthropists for investments in research and novel therapies, and bringing the patient voice to academic committees, conferences, and research initiatives [19,20,21,22,23]. Up until 2018, there were no patient-led organizations dedicated exclusively to advocating for AYA cancer in Canada.

In 2018, a group of AYA patient advocates came together and established “AYA Canada” (later renamed “AYA Can”)—the first national peer-led patient advocacy organization in Canada dedicated to improving the experiences and outcomes for AYAs with cancer. AYA Can is a peer-led national organization advocating for AYAs living with and beyond cancer. The aim of this article is to describe the development, activities, impact, and future directions of AYA Can.

## 2. The Idea for a Canadian Peer-Led Advocacy Organization in AYA Cancer

“AYA Canada” (later renamed “AYA Can”) was founded in 2018 when two AYA cancer survivors, B. Garand Sheridan (Manitoba) and J. Deleemans (Alberta), met at an AYA National Network meeting hosted by the Canadian Partnership Against Cancer (CPAC). During this meeting, gaps in AYA cancer research and care in Canada were highlighted, and possible strategies to bridge the gaps were explored. One key gap identified was the lack of a coordinated, patient-led advocacy effort for AYA cancer in Canada. Representatives from other jurisdictions (e.g., Canteen in Australia [24]) discussed the great progress that emerged for AYA cancer resources and outcomes as a result of organized patient-led advocacy and met with B. Garand Sheridan and J. Deleemans. At the time, while organizations existed in Canada to provide programming and support for AYA patients with cancer, there was no group dedicated solely to raising awareness of and advocating for the unique needs of AYAs and encouraging changes in health care and policy. As a result of these conversations, B. Garand Sheridan and J. Deleemans came together to establish Canada’s first peer-led advocacy organization for AYA Cancer in Canada.

## 3. Building the Team

Following the CPAC meeting, B. Garand Sheridan and J. Deleemans connected with M. Lang (Alberta), another AYA patient advocate, and the three comprised the initial leadership of AYA Canada. Their efforts started by establishing a community of practice comprised of healthcare professionals, researchers, charitable organizations, and patients with an interest in AYA advocacy in Canada. They then started hosting quarterly virtual calls (later referred to as “Community Calls”) with this community of practice to refine the priorities of AYA Canada (more information in the Initiatives section below).

As the community of practice grew, additional patient advocates with lived experience of AYA cancer joined the leadership of AYA Canada, including C. Thurston (Manitoba), M. Tomeh (Ontario), E. Piercell (Ontario), J. Gisser (Manitoba), D. Pike (British Columbia), and V. Ramasamy (Ontario). Between the patient advocates, the AYA Canada leadership had expertise in medical science, research, accounting, law, finance, communications, community outreach, and activism, which helped to propel the organization forward. All board members serve in a volunteer capacity, and many balance this role alongside other career, medical, and family demands. Between 2020–2023 the composition of the leadership team fluctuated between four to six members at a time as individuals encountered changes to their health and availability. C. Thurston was appointed as Chair of the leadership team in February 2021 and has held that role since.

**Figure 1:**
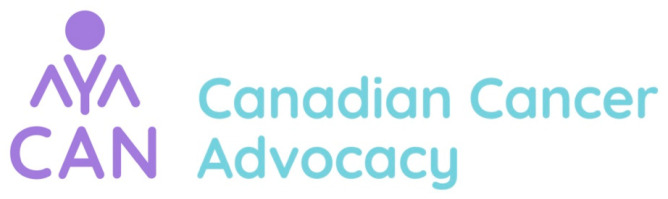
AYA Can—Canadian Cancer Advocacy Logo.

When the COVID-19 pandemic struck in March 2020, progress in the growth of AYA Canada was slowed. Nonetheless, the community of practice virtual calls continued. In September 2020, the team registered for an organization email. Social media accounts on platforms including Twitter (@AYACan_Cancer), Facebook (@AYACan.Cancer), Instagram (@AYACan_Cancer), and LinkedIn (@AYA CAN—Canadian Cancer Advocacy) were established in March 2021. A logo was created in August 2021 (Figure 1), and a website (www.ayacan.ca) was launched in March 2022.

In September 2021, the leadership changed the name of the organization from “AYA Canada” to “AYA Can—Canadian Cancer Advocacy” and registered as a non-profit corporation. An organizational mission was established. Next, the AYA Can leadership team (now an official “Board of Directors”) initiated an application to become a registered charity with the Canadian Revenue Agency. AYA Can was granted its formal charity status on 1 January 2023.

## 4. AYA Can Activities and Initiatives

**Figure 2:**
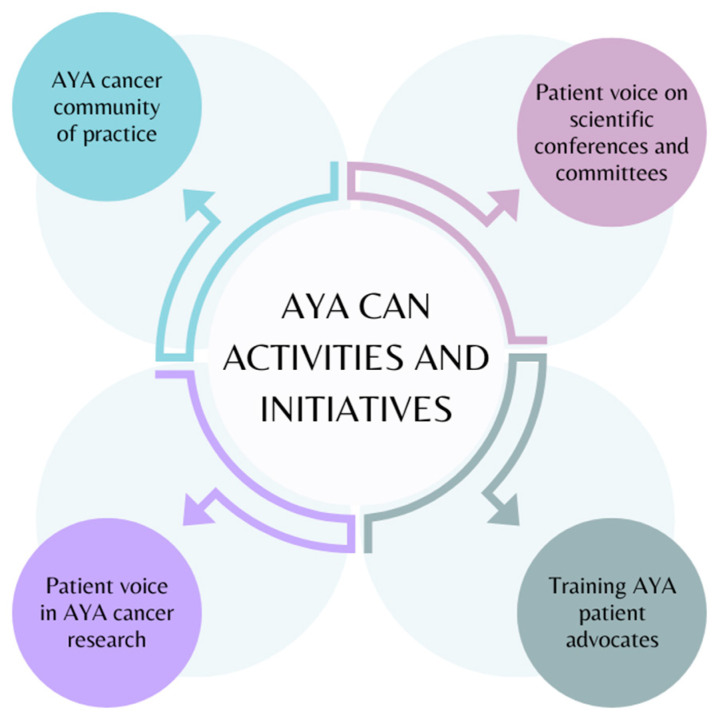
Summary of AYA Can—Canadian Cancer Advocacy Activities and Initiatives.

Over the last 5 years, AYA Can has been engaged in numerous activities to advance its mission of advocating to improve the experiences and outcomes of AYA patients with cancer. AYA Can’s activities have included connecting patients, healthcare providers, researchers, and charitable organizations through a community of practice, advocating for the needs of AYAs at academic conferences and on scientific committees, collaborating with scientists to advance AYA cancer research, and training the next generation of AYA patient advocates. These initiatives are summarized below and in Figure 2.

### 4.1. Building a Community of Practice in AYA Cancer

One of AYA Can’s main objectives has been to establish and maintain a robust community of practice comprised of patients, healthcare providers, researchers, and representatives from charitable organizations who are dedicated to advocating for AYA cancer in Canada. This community of practice overcomes historical fragmentation in the Canadian AYA cancer community by bringing together diverse individuals and organizations in the AYA cancer space. The goal is to facilitate communication and sharing of resources and opportunities among AYA advocates in Canada to ultimately improve outcomes.

Establishing a community of practice was one of AYA Can’s first activities led by the initial leadership. In 2019, the early leadership began meeting with relevant individuals and organizations to introduce the concept of the organization and explore opportunities for collaboration. This practice has been continued by the current Board Chair, who regularly meets with AYA cancer advocates in Canada and invites them to join the community through subscribing to the newsletter and attending the community calls.

#### 4.1.1. Newsletters

AYA Can distributes a monthly newsletter by email to a roster of over 275 individuals in the community of practice. The newsletter encompasses content including updates on the work of AYA Can, opportunities to participate in AYA cancer research, and community events and programs relevant to the AYA community that are looking for participants as well as programs available to patients. This way, any program leads can share this with their patient lists, and others can share the research they are undertaking and how it can be beneficial to each other.

#### 4.1.2. Community Calls

To date, AYA Can has hosted 15 virtual meetings (known as “Community Calls”) that bring together a community of practice of AYA advocates in Canada. The first community call was held in October 2019, and since then, these gatherings have been held quarterly. The average call attendance is 24 individuals (range: 9–30) and includes a mix of patients, caregivers, healthcare providers, researchers, and representatives from charitable organizations. During the calls, members of the community of practice are provided with the opportunity to deliver updates on programs, initiatives, and research to the AYA cancer community. Opportunities for collaboration, research participation, and outreach are highlighted. The community calls have resulted in improved communication and collaboration for AYA initiatives in Canada.

### 4.2. Bringing the AYA Patient Voice to Scientific Conferences, Meetings, and Committees

Members of the AYA Can Board regularly deliver presentations at local (e.g., Princess Margaret Hospital AYA Virtual Symposium), national (e.g., American Association of Cancer Research Annual Meeting, Canadian Cancer Clinical Trials Network Annual Stakeholder Meetings, Colorectal Cancer Resource & Action Network Early Onset Cancer Virtual Symposium) and international (e.g., World Congress of Psycho-Oncology, Society for Integrative Oncology Annual Conference) meetings. The objectives of the presentations are to share the AYA patient experience with researchers and administrators, advocate for the unique needs of AYAs, and disseminate information on the work of AYA Can. For instance, the presentation entitled “AYA CAN: Canadian Cancer Advocacy—A Pan-Canadian Patient Led Organization for Adolescent and Young Adults” was delivered at the World Congress of Psycho-Oncology and focused on describing the establishment and activities of the organization.

AYA Can has also been invited to serve on various academic and administrative committees to represent the AYA perspective in cancer research and care. Such committees have included the BiocanRX Learning Institute Working Group, the Canadian Association of Psychosocial Oncology Advocacy Committee, the Canadian Cancer Clinical Trials Network Management Committee, the Advancing Childhood Cancer Experience, Science & Survivorship Psychosocial and Survivorship Matrix Leadership Committee, the AYA Cancer Priority Setting Partnership Steering Committee, and a provincial Steering Committee to advance oncofertility services in British Columbia and the Yukon.

### 4.3. Bringing the AYA Patient Voice to Cancer Research

AYA Can has been at the forefront of patient engagement in cancer research in Canada. Since 2020, AYA Can has helped to advance research for AYA cancer in Canada by providing letters of support for seven grant applications submitted by Canadian investigators. As an organization, AYA Can is also co-leading the AYA Cancer Priority Setting Partnership (PSP) with researchers at the University of Calgary. The AYA Cancer PSP is a national James Lind Alliance [25] priority-setting initiative that will identify the Top 10 research priorities for AYA cancer in Canada. This initiative will shape the future of AYA cancer research in the country by establishing a patient-oriented research agenda for AYA cancer that is primed for action by researchers, funding agencies, and policymakers (for more information, see [26]). Additionally, members of the AYA Can Board have served as patient partners on several research projects across the country, embedding the AYA patient voice across all aspects of the research process.

### 4.4. Training the Next Generation of AYA Patient Advocates

To build capacity within AYA cancer advocacy in Canada, AYA Can has developed a novel “patient ambassador” program. The goal of the program is to train and empower AYA patients with the knowledge and skills they need to advance the mission of AYA Can. The AYA Can Board of Directors meets with the patient ambassadors quarterly. These meetings involve presentations and discussions of relevant content (e.g., introduction to advocacy, patient engagement, and the research process) and opportunities. AYA Can patient ambassadors may be matched with opportunities that fit with their skills and interests to advocate for AYA research and care in Canada (e.g., serving on a committee and partnering on research projects). There are currently 67 AYAs engaged in the patient ambassador program, representing diverse voices from across the country.

## 5. Future Directions for AYA Can

AYA Can has made great strides towards advancing advocacy efforts for AYA cancer in Canada over the last 5 years. Several initiatives have been identified as priority areas for future advocacy efforts. AYA Can is currently operated entirely by volunteer patients with no external funding. The scale, spread, and sustainability of the organization’s activities will require an investment of funds to support its operations, plans for which are currently being explored. To date, AYA Can has not had a team member dedicated to fundraising or with specialized expertise in this area. However, recruitment efforts for additional team members have focused on the need for this skill set. Funding has recently been secured through small philanthropic donations to support AYA Can with developing a national “Resource Hub.” The resource hub will be housed on the new AYA Can website and will serve as a tool to guide AYA patients and healthcare providers navigating cancer in Canada. It will also democratize scientific knowledge on AYA cancer (i.e., make information more easily accessible and understood) for all members of the community. The hub will be a central repository for active research opportunities for AYAs, contacts for provincial AYA programs, available community services and supports for AYAs that can be filtered by location and cancer type, accessible and up-to-date data on AYA cancer in Canada to support advocacy efforts, and more. Development of the resource hub is currently underway.

Future directions for AYA Can also include plans to petition provincial and national governments and funding agencies for financial investments to improve AYA research and care in Canada. Efforts will focus on the development of research competitions to advance the research priorities identified through the ongoing AYA Cancer Priority Setting Partnership and broader goals brought forward by the AYA Can community.

## 6. Conclusions

This article describes the development and impact of AYA Can, a peer-led national organization advocating for improved experiences and outcomes for individuals affected by adolescent and young adult (AYA) cancer in Canada. Since 2018, AYA Can has become a leading voice for AYA cancer in Canada through its work establishing a community of practice, advocacy on national meetings and committees, engagement in AYA cancer research, training of AYA patient advocates, and delivery of information and knowledge. The work of AYA Can has improved collaboration among the AYA cancer community, supported research teams with securing funding for AYA cancer research, and amplified AYA patient priorities on a national scale, making crucial progress towards improving experiences and outcomes for AYA cancer in Canada. While AYA Can has made great progress advocating AYA cancer research and care in Canada, investments are needed from national entities to continue to meaningfully push the AYA cancer agenda forward.

## Data Availability

No new data were created or analyzed in this study. Data sharing is not applicable to this article.
